# Glymphatic system dysfunction in central nervous system malignancies: A narrative review

**DOI:** 10.1007/s10143-026-04508-2

**Published:** 2026-10-02

**Authors:** Gabriela Villacis, Justus C. Rudolf, Claudia Ditz, Peter Schramm, Patricia Ulloa

**Affiliations:** 1 Department of Neuroradiology, University Hospital Schleswig-Holstein (UKSH), Luebeck, Germany; 2 Department of Neurosurgery, University Hospital Schleswig-Holstein (UKSH), Luebeck, Germany

**Keywords:** Glymphatic system, Neurofluids, Brain waste clearance, Brain tumors, Magnetic resonance imaging, DTI ALPS index, CNS malignancies

## Abstract

The hypothesis of the glymphatic system as playing an important part in clearing waste from the brain has revolutionized our understanding of cerebrospinal and interstitial fluid dynamics in the brain. This review explores current knowledge of central nervous system (CNS) malignancies and their implications in the glymphatic system function. Here, we synthesize all available preclinical and clinical evidence on glymphatic dynamics in CNS malignancies. The human data evaluated (gliomas, meningiomas, peritumoral edema, and cerebral metastasis) constitute the total scope of current research. We conducted a systematic search in the PubMed database for studies published between 1 January 2015 and 1 January 2026. We selected 22 studies based on their relevance regarding brain tumors and their relationship to glymphatic system dysfunction. In contrast to animal studies that rely on contrast-enhanced imaging, human studies predominantly use the diffusion tensor imaging along perivascular spaces (ALPS) index due to its noninvasive nature and feasibility with multidirectional diffusion-weighted imaging, which is available on most clinical scanners. However, interpretation of the findings remains the subject of debate, particularly regarding the influence of microstructural factors and other parameters on index values. Consequently, current approaches increasingly assess the ALPS index as a potential indirect marker of glymphatic function alongside complementary MR-based techniques, including multiparametric mapping, volumetry, free water estimation, and DTI-derived microstructural measures. Further research in CNS tumors is required to clarify consistent patterns and address the variability of reported findings.

## Introduction

**Fig. 1:**
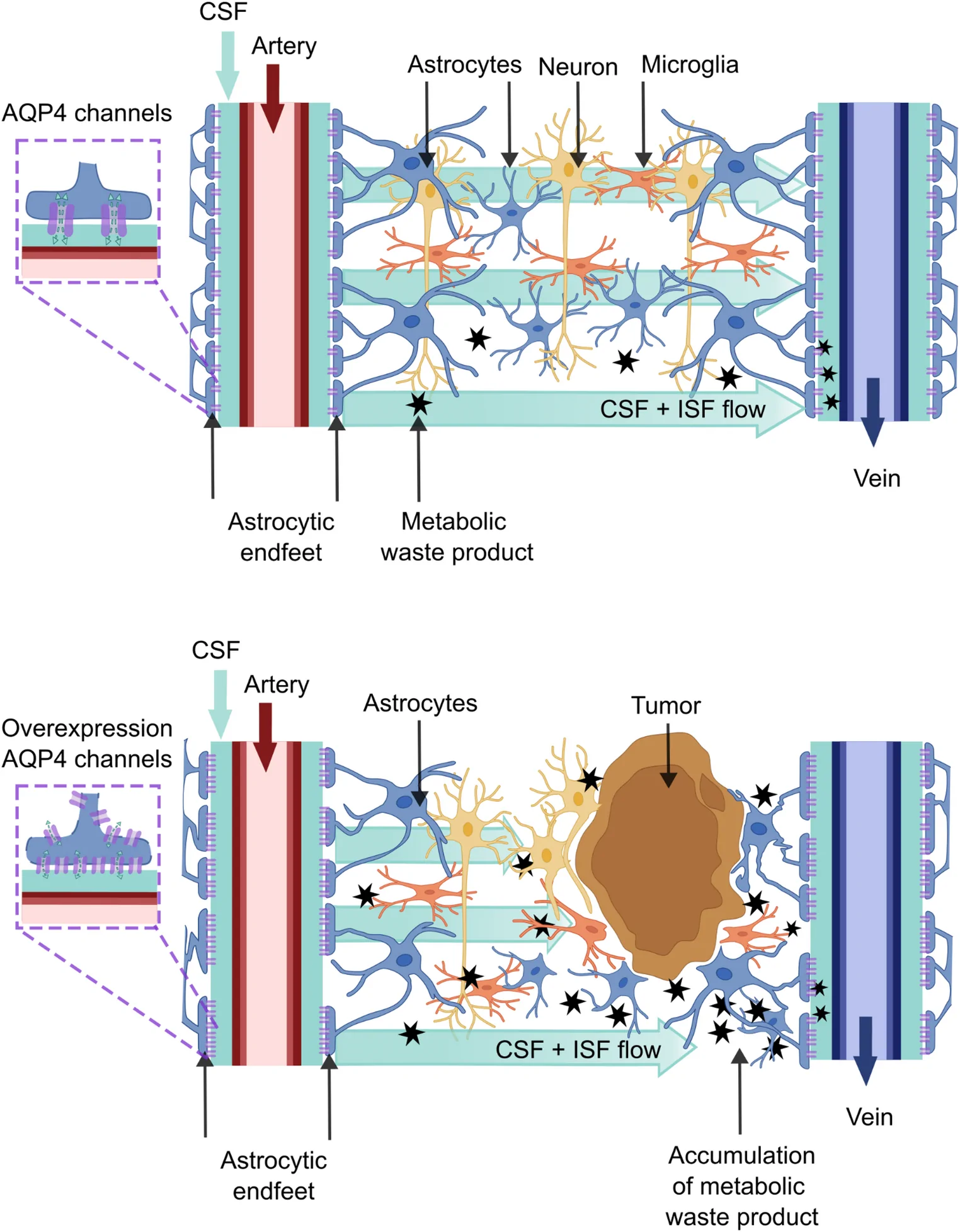
Illustration of glymphatic system architecture. (**A**) The physiological glymphatic system. Flow of cerebrospinal fluid (CSF) is mediated by aquaporin 4 channels (AQP4) at the endfeet of astrocytes, flowing from the arterial end, through the parenchyma, where the CSF-ISF exchange takes place, into the perivenous spaces, where the waste products are washed out of the brain. (**B**) Brain tumor–related glymphatic impairment. Cell debris and accumulation of other waste products obstruct neurofluid flow and adjacent brain tissue is compressed by the tumor. AQP4 is overexpressed, now being present along the whole astrocyte membrane and leading to an abnormal neurofluid flow and possibly to edema. Consequently, waste products cannot be removed efficiently from the brain. Created in BioRender. Ulloa, P. (2026) https://BioRender.com/uwx84tb

The recent recognition of the glymphatic system (GS) has revolutionized our understanding of waste clearance in the brain [1], as for many years, the lack of a “brain lymphatic system” has raised questions on how the brain cleanses itself. The term “glymphatic” comes from the combination of “glial” and “lymphatic” [2], highlighting the parallel with the body’s lymphatic system. The GS is active during sleep and is dependent on aquaporin 4 (AQP4) in the endfeet of astrocytes, arterial pulsation [3], breathing, and pressure gradients. The role of AQP4 channels is critical for correct exchange between cerebrospinal fluid (CSF) and interstitial fluid (ISF). Indeed, AQP4 overexpression in glioma has been identified as a prognostic factor [4] and can damage the blood-brain barrier (see Fig. 1), impairing ISF outflow and contributing peritumoral edema [5].

The GS describes the CSF flow from the arterial vessels through the interstitial space, mixing with ISF, and then exiting the brain parenchyma along the venous perivascular spaces [3, 6, 7], carrying out toxic waste products. While initially studied in neurodegeneration, the GS is now recognized as a critical stakeholder in the pathophysiology of brain malignancies, including gliomas, meningiomas, brain metastases, and retinoblastoma. Brain malignancies can profoundly disturb the local environment, through mass effect, vascular remodeling, peritumoral edema, and changes in intracranial pressure, all of which may interfere with GS function. Several studies have shown that the GS may play an important role in brain tumor growth, immunity, and therapy [8]. Here, the development of the diffusion tensor imaging (DTI) along perivascular spaces (ALPS) index [9] has opened a window to indirectly assess GS function. Current research has consistently correlated a low ALPS index, as a predictor of shorter overall survival, and cognitive decline. Emerging evidence suggests that the alterations in brain fluid dynamics are not limited to the side harboring the tumor, but also affect the contralateral hemisphere, implying a global breakdown in neurofluid homeostasis and thereby posing risk to the still unharmed tissue, which then causes secondary impairment.

This literature review aims to cover current knowledge of the GS and brain malignancies. Here, we will highlight which techniques have been used to evaluate GS function and present common elements in research and contradictions, which should result in greater motivation to continue this branch of research.

## Methods

A systematic search was conducted in PubMed to identify studies addressing the GS in the context of central nervous system (CNS) tumors. The search strategy combined the term “glymphatic” in the Title/Abstract field with tumor-related keywords as follows: (tumor OR cancer OR glioma OR glioblastoma OR meningioma OR metastasis OR retinoblastoma OR chemotherapy). Studies were excluded using the NOT operator for titles containing terms unrelated to the scope of this review: (sleep, dementia, Parkinson, Alzheimer, eye, trauma, traumatic brain injury, aged, aging, hydrocephalus, blast). The search (1 January 2015–1 January 2026) yielded 67 publications. Limiting results to English-language articles and excluding preprints and case reports resulted in 56 studies for screening.

Following title and abstract assessment, 34 articles (23 review papers and 11 original studies) were excluded because the GS and/or CNS tumors were only briefly mentioned and were not the primary focus of the work. The final selection comprised 22 original research articles, which were included in this review.

## Results

Previous review articles have covered the topic of gliomas [10–12], brain tumor treatment [13], and general GS with partial implications in brain tumors [2, 14]. Here, we cover animal models, computer simulations (Table 1) and CNS tumors (Table 2).

**Table 1:** Papers that included animal experiments and simulations. Abbreviations: MRI: magnetic resonance imaging, CE: contrast-enhanced, IDH1: isocitrate dehydrogenase 1

Topic	Author		Technique
Tumor mechanical forces	Siri et al. [15] (2024)	Model	Biomechanical modeling
Glioblastoma	Kaur et al. [16] (2023)	Rats	MRI dynamic CE and kinetic modeling
Pituitary tumor	Li et al. [17] (2023)	Rats	MRI dynamic CE, kinetic modeling and T2-weighted
Glioma	Xu et al. [18] (2021)	Rats	MRI post CE, T1, T2, and T1 mapping, intracranial pressure sensor (glioma)
Mutant IDH1	Pickard et al. [19] (2016)	Mice	Perfusion microdialysis and liquid chromatography-mass spectrometry

**Table 2:** Papers on human research/experiments. Abbreviations: MRI: magnetic resonance imaging, ALPS: along perivascular spaces index, CE: contrast-enhanced; MISL: magnetization transfer indirect labeling

Topic	Author	Technique
General brain tumors	Villacis et al. [20] (2024)	MRI ALPS
	Gao et al. [21] (2024)	MRI ALPS, multiparametric mapping
Peritumoral edema	Turkin et al. [22] (2023)	MRI ALPS (T1, T2, T2-FLAIR, DWI, post/contrast T1)
Edema in meningioma	Turkin et al. [23] (2023)	CT and MRI ALPS (T1, T2, T2-FLAIR, DWI, post/contrast T1)
	Toh et al. [24] (2021)	MRI ALPS
Edema in cerebral metastasis	Toh et al. [25] (2021)	MRI ALPS, CE perfusion
Retinoblastoma	Deike-Hofmann et al. [26] (2022)	MRI CE
Glioma		
	Lie et al. [27] (2025)	MRI ALPS
	Ono et al. [28] (2025)	MRI ALPS
	Hagiwara et al. [29] (2025)	MRI ALPS + free water
	Fang et al. [30] (2025)	MRI ALPS + metrics
	Tian et al. [31] (2025)	MRI ALPS
	Liang et al. [32] (2025)	MRI ALPS + volumetric
	Wang et al. [33] (2025)	MRI ALPS
	Zeng et al. [34] (2025)	MRI ALPS
	Zhu et al. [35] (2023)	MRI ALPS
	Toh and Siow [36] (2021)	MRI ALPS

### Animal experiments and simulations

Animal experiments play a vital role in advancing our understanding of the complex mechanisms underlying the GS and its involvement in brain diseases. Initially, Pickard et al. [19] studied in mice the distribution of D-2-hydroxyglutarate (D2HG) in mutant isocitrate dehydrogenase 10 (IDH1) brain tumors. Although they did not find a significant effect in the glymphatic clearance of the D2HG fluid, they provide evidence that D2HG exits glioma cells and is present in the ISF. Recent investigations have highlighted the role of glymphatic dysfunction using contrast-enhanced magnetic resonance imaging (CE-MRI) in the context of glioma [16, 18] and pituitary tumors [17]. These studies observed that CSF influx and clearance from the brain were hindered in rodent models. This finding suggests that brain tumors reduce efficiency in removing metabolic byproducts, linking this to a GS dysfunction. Additionally, computer simulations showed that tumor-induced mechanical forces compress the perivascular spaces, thereby disrupting the normal function of brain waste clearance [15]. Overall, these studies underscore the significance of glymphatic fluid dynamics in the context of brain tumors and the possible implications for understanding tumor biology.

### Human research

**Fig. 2:**
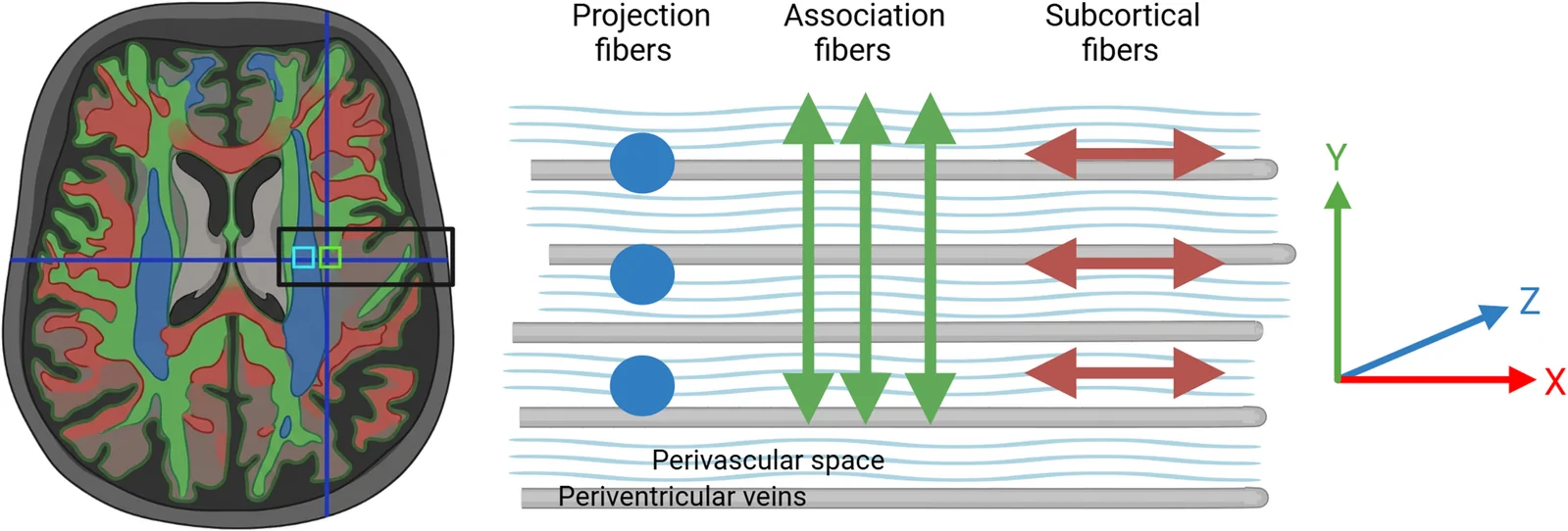
Schematic explanation of the principle underlying the diffusion-tensor imaging analysis along the perivascular space (ALPS) method. (**A**) At the lateral ventricular body level, the neural fibers are identified on a susceptibility-weighted image. (**B**) Regions-of-interest are placed on a color-coded fractional anisotropy map. (**C**) Perivascular water flows perpendicular to projection and association neural fibers. Therefore, diffusivity along the x-axis at these areas indirectly reflects perivascular glymphatic flows. Created in BioRender. Ulloa, P. (2026) https://BioRender.com/fxqxzrx

Human research in this field is almost exclusively based on MRI, with only one study adding computed tomography (CT). All 18 reviewed research articles propose the ALPS index as an indirect biomarker for GS function. According to Taoka et al. [9], the ALPS index may offer a first glance of the status of the GS status by using water movement along the periventricular veins as an indirect measure of waste clearance function (see Fig. 2). The ALPS index has become very popular in the last years due to its noninvasive nature and simplicity. The index is calculated as a diffusivity ratio from the diagonal elements of the diffusion tensor matrix, serving as an indirect representation of water diffusion within the perivascular spaces of the medullary veins. These veins are ideal for this measurement because they are parallel to each other and perpendicular to the ventricle wall. By placing regions-of-interest in the projection and association areas, researchers could theoretically isolate the diffusion tensor elements perpendicular to the medullary veins to calculate the index. This value has been used to indirectly infer the state of the brain’s GS function, where the accumulation of metabolic waste and misfolded proteins in the perivascular space can hinder CSF-ISF flow dynamics, resulting in a lower ALPS index relative to healthy controls (HC). However, current research is putting the meaning of the ALPS index into question since it is strongly influenced by white matter microstructure.

#### Human brain tumors

Research on brain tumors by Villacis et al. [20] and Gao et al. [21] analyzed patients with various tumor types alongside age- and sex-matched HCs using the ALPS index. Both studies found lower ALPS index values in patients than in HC, which they related to impaired GS function. However, Villacis et al. [20] noted that the ALPS index was significantly higher in the contralateral hemisphere than in controls, suggesting a possible compensatory mechanism within the GS. Gao et al. [21] further explored the relationship between the ALPS index and local tumor environment factors, including AQP4 expression, PTBE volume, and tissue compression. This work offers new insights into the mechanisms of PTBE and provides a new approach to study the GS using multiparametric MRI.

#### Peritumoral brain edema

The formation and development of peritumoral brain edema (PTBE) have been investigated in the context of the contribution of the GS, with researchers utilizing the ALPS index as an indirect proxy to evaluate GS function [22–25]. These studies generally associate PTBE with GS dysfunction, as suggested by a lower ALPS index in patients vs. HC, implying that impaired waste clearance may contribute to edema formation across various tumor types. Furthermore, the authors generally found that the ALPS index in the contralateral hemisphere remained similar to that of HCs, suggesting that the low ALPS index in the tumor-bearing side could be related to GS impairment. The primary discrepancies between these studies arise from the statistical relationship between the ALPS index and physical edema volume. In meningioma patients, Toh et al. [24] and Turkin et al. [22] found that the ALPS index is significantly associated with PTBE volume. However, Toh et al. [25] observed a critical distinction in brain metastases: while the ALPS index was reduced, it did not statistically correlate with PTBE volume. This suggests that edema in metastatic lesions may be driven by microvessel permeability rather than the drainage obstruction that dominates meningiomas. Additionally, Turkin et al. [23] identified unique morphological predictors specifically for meningiomas, noting that uneven contours and large volume are significant risk factors for edema. Additionally, they observed that a “CSF cleft sign” may act as a protective factor, significantly reducing the risk of PTBE development. However, these differences likely reflect variations in study design, tumor biology, and the distinct microstructural factors inherent to different tumor entities.

#### Glioma

Glioma is the most extensively studied CNS tumor, and all the cited studies based their glymphatic research on MRI and the ALPS index. The general aim is to examine the ALPS index and its relationships with clinical factors such as tumor grade, IDH1 mutation, and tumor volume (with or without PTBE). Several studies (Li et al. [27], Tian et al. [31], and Wang et al. [33) suggest the ALPS index is a potential biomarker to predict survival rate. Hagiwara et al. [29] further enhanced IDH-wildtype prognosis by using free-water imaging in the contralateral hemisphere to quantify extracellular fluid stagnation, while Ono et al. [28] linked low ALPS index in the same hemisphere to postoperative cognitive scores, suggesting that diffusion disruption could be associated with impaired glymphatic clearance. Fang et al. [30] and Zhu et al. [35] refined the diagnostic utility for grading and IDH status using multi-metric DTI models, while Toh et al. [25] identified peritumoral edema as an independent driver of glymphatic stasis in brain metastasis. In a mechanism-based approach, Liang et al. [32] provided evidence of CSF retention, supporting the drainage impediments described by Zeng et al. [34]. However, findings regarding the ALPS index in high-grade gliomas are contradictory: Fang et al. [30] found that the contralateral hemisphere had lower index, whereas Tian et al. [31] and Wang et al. [33] observed lower values ipsilaterally. Similarly, the impact of tumor mass on the index is debated, with Liang et al. [32] finding a correlation between the index and tumor and edema volumes, that Toh and Siow [36] and Tian et al. [31] did not. Overall, most studies agree that glioma patients have a significantly lower ALPS index in tumor-bearing hemispheres compared to the contralateral side and HC (with Fang et al.^30^ as an exception), possibly reflecting more severe glymphatic impairment in high-grade and IDH-wildtype tumors.

## Discussion

This narrative literature review synthesizes current evidence on GS function in CNS cerebral malignancies, where the most used technique in clinical research is the MR-based ALPS index. We identified common ground and discrepancies in the published results. A consistent finding across studies is a reduced ALPS index in patients compared to HC. This has been interpreted as indirect evidence of glymphatic dysfunction, potentially contributing to impaired waste clearance, cognitive impairment and PTBE. However, this interpretation is complicated by multiple confounding factors that are often inadequately controlled or discussed in literature.

Clinically, the ALPS index value is strongly influenced by tumor biology, being the changes in microstructure the most important cofounder [37–41]. High-grade gliomas [32], IDH-wildtype tumors [27], and PTBE [31] consistently show lower ALPS values than HC. However, this may reflect not only glymphatic impairment but also the extent of tumor infiltration, necrosis, and PTBE itself, all of which alter tissue microstructure and water diffusion. Additionally, PTBE may arise from microvascular leakiness (e.g., in metastases) rather than drainage obstruction, suggesting that low ALPS in such cases may reflect edema-related tissue changes rather than primary glymphatic failure. Furthermore, while most studies agree that glioma patients have significantly lower ALPS index in the tumor-bearing hemisphere compared to contralateral side and HC, the impact in tumor grade and IDH-status is uncertain [33]. This may suggest changes in whole brain diffusion across tumor types. Also, compensatory mechanisms, such as increased activity in unaffected areas or alternative drainage pathways, could also contribute to these findings. Moreover, changes in the ALPS index considering the patient’s treatment history such as surgery, radiotherapy, and chemotherapy. These treatments can independently affect the ALPS values by altering local tissue architecture or inducing fibrosis and demyelination. These factors complicate the interpretation of ALPS as a pure biomarker of glymphatic function. Other discrepancies likely arise from the different study designs (e.g., sample size, patient demographics, and exclusion parameters), data analysis methods, and the specific diseases being studied. Therefore, future studies should direct attention to selecting larger sample sizes, well-defined patient cohorts.

Technically, the ALPS method has several limitations. It estimates diffusivity along the perivascular space in a small region-of-interest, parallel to the medullary veins. This extrapolation assumes that the whole-brain glymphatic function can be inferred from this small area in deep white matter. Meanwhile, amyloid β and tau proteins are produced and accumulate in the brain cortex [42]. Additionally, the assumption that perivascular spaces run parallel to the x-axis at the lateral ventricular body may also be violated in these patients due to tumor-induced mass effect, ventricular distortion, or white matter disruption. Furthermore, methodological variability across studies introduces significant heterogeneity and prevents direct ALPS index comparison between studies [43]. Differences between scanners, magnetic field strength and DTI protocol (b-values, number of directions) directly affect signal-to-noise ratio, tensor accuracy, and the stability of the ALPS index. Furthermore, inconsistent ROI placement, ranging from manual drawing to fixed voxel cubes or template-based methods, introduces inter-study and inter-reader variability [43]. To address this issue automatic approaches have been suggested to quantify the water diffusivity in the periventricular area [44, 45]. Hence, standardized imaging acquisition and imaging analysis methods are important for further research.

These confounders collectively limit the interpretability of ALPS as a direct proxy for glymphatic function and undermines the validity of the measurement. While reductions in ALPS are consistently reported in tumor patients, they likely reflect a composite signal of structural, inflammatory, vascular, and treatment-related changes rather than isolated glymphatic dysfunction. Therefore, should not be directly linked to GS function [46].

## Conclusion

In summary, while the DTI-ALPS index holds potential as a non-invasive biomarker for glymphatic dysfunction in CNS malignancies, its clinical and research utility is constrained by significant confounding factors. These include both clinical variables (tumor grade, molecular subtype, edema mechanism, treatment history) and technical parameters (ALPS index methodology and ROI placement, MRI parameters, anatomical distortion) that must be rigorously controlled and reported. Therefore, the ALPS index does not solely represent diffusion in the perivascular space, but also other processes, such as axonal degeneration [40]. Hence, changes in the ALPS index should be interpreted with caution [40, 47, 48]. To address these limitations, future studies should adopt standardized protocols and incorporate advanced microstructural imaging (e.g., free water imaging, diffusion kurtosis imaging, multishell diffusion weighting) to decouple the effects of tissue characteristic from GS clearance and multimodal imaging (e.g., multiparametric MR, arterial spin labeling). Also, longitudinal study designs will help to disentangle true glymphatic impairment from confounding influences. Only then can ALPS be reliably translated into a clinically meaningful tool for prognosis, treatment monitoring, and mechanistic insight in neuro-oncology.

## Data Availability

No datasets were generated or analysed during the current study.
